# A mathematical model for the within-host (re)infection dynamics of SARS-CoV-2

**DOI:** 10.1016/j.mbs.2024.109178

**Published:** 2024-05

**Authors:** Lea Schuh, Peter V. Markov, Vladimir M. Veliov, Nikolaos I. Stilianakis

**Affiliations:** a Joint Research Centre (JRC), European Commission, Via Enrico Fermi 2749, Ispra, 21027, Italy; b London School of Hygiene & Tropical Medicine, University of London, Keppel Street, London, WC1E 7HT, United Kingdom; c Institute of Statistics and Mathematical Methods in Economics, Vienna University of Technology, Wiedner Hauptstraße 8-10, Vienna, 1040, Austria; d Department of Biometry and Epidemiology, University of Erlangen–Nuremberg, Waldstraße 6, Erlangen, 91054, Germany

**Keywords:** COVID-19, Infectious disease modeling, Viral kinetics, Immune response, Reinfection, Virus variants

## Abstract

Interactions between SARS-CoV-2 and the immune system during infection are complex. However, understanding the within-host SARS-CoV-2 dynamics is of enormous importance for clinical and public health outcomes. Current mathematical models focus on describing the within-host SARS-CoV-2 dynamics during the acute infection phase. Thereby they ignore important long-term post-acute infection effects. We present a mathematical model, which not only describes the SARS-CoV-2 infection dynamics during the acute infection phase, but extends current approaches by also recapitulating clinically observed long-term post-acute infection effects, such as the recovery of the number of susceptible epithelial cells to an initial pre-infection homeostatic level, a permanent and full clearance of the infection within the individual, immune waning, and the formation of long-term immune capacity levels after infection. Finally, we used our model and its description of the long-term post-acute infection dynamics to explore reinfection scenarios differentiating between distinct variant-specific properties of the reinfecting virus. Together, the model’s ability to describe not only the acute but also the long-term post-acute infection dynamics provides a more realistic description of key outcomes and allows for its application in clinical and public health scenarios.

## Highlights

• A novel model of the within-host SARS-CoV-2 infection dynamics.
• Describes acute infection dynamics of individual viral load data accurately.
• Reveals long-term post-acute SARS-CoV-2 infection dynamics.
• Accommodates reinfections accounting for higher infectivity or immune escape variants.

## 1 Introduction

The interactions between severe acute respiratory syndrome coronavirus type 2 (SARS-CoV-2) and the immune system during infection are complex. Viral load measurements of the upper respiratory tract provide a quantitative method to capture the within-host SARS-CoV-2 infection dynamics [1]. To dissect and quantify the underlying pathogenic mechanisms mathematical models have been developed, incorporating features such as viral loads, time of clinical symptom onset or infectiousness [2].

Viral within-host dynamics are commonly described by the target-cell limited (TCL) model [3]. The TCL model describes the dynamics of susceptible (target) cells, infected cells, and free virus populations during the course of an infection. In the past few years, TCL-based models have been employed to also describe the within-host infection dynamics of SARS-CoV-2. Early efforts focused on quantitatively describing the measured within-host viral load dynamics in individuals [4], [5] and on linking viral loads to infectiousness [6], [7]. These models differentiated between infectious and non-infectious virus, such as residual viral genome fragments, or by adding simple, often non-mechanistic descriptions of the human immune response [4], [5], [6], [7]. Later models integrated more mechanistic formulations of the human immune response and quantitatively compared different immune response effects and targets [8], [9]. These models provided further insights into SARS-CoV-2 infection dynamics, revealing heterogeneity in the infectiousness levels between individuals [8] and suggesting differences in viral dynamics between variants [9], [10]. TCL-based models of SARS-CoV-2 infection dynamics have lately also been used to describe the viral load dynamics during antiviral treatment and, consequently, to obtain insights into phenomena such as viral rebound and resistance formation [11], [12]. To date, models describing the within-host SARS-CoV-2 infection dynamics typically focus on the short-term acute phase of an infection. The assumptions that these models make are often at odds with simple but highly relevant and important clinical realities, such as the re-establishment of susceptible cells to pre-infection levels, full and permanent clearance of the infection from the individual, the corresponding decline of the immune response, and the associated established long-term post-acute infection immunity levels after exposure and infection. Only a few models account for a re-establishment of susceptible cells by including a constant [5], [13], [14] or logistic production term [12]. Other models ensure a full and permanent clearance of the infection by manually setting the viral production rate to zero for viral loads below a minimal viral threshold [15] or consider the viral dynamics for periods up to 7 weeks post infection [15], [16]. To the best of our knowledge, there exists no within-host SARS-CoV-2 model accounting for all of the above described long-term post-acute infection effects simultaneously. A more complete representation of the overall infection process, however, is required to study long-term effects and link primary and reinfection events.

Here, we present a within-host SARS-CoV-2 model, which offers a more realistic account of the underlying infection dynamics by recapitulating and linking the acute short-term and the expected long-term post-acute infection dynamics. These include the re-establishment of the number of susceptible epithelial cells to the initial pre-infection level, a full and permanent clearance of the infection from the individual, the formation of long-term post-acute infection immune capacity levels after infection, and the waning of immunity. Finally, we demonstrate that due to the long-term post-acute description, the model can accommodate reinfection events, taking into account differences in infectivity and levels of immune escape of the reinfecting virus variant.

## 2 Results

### 2.1 Within-host SARS-CoV-2 model

**Fig. 1:**
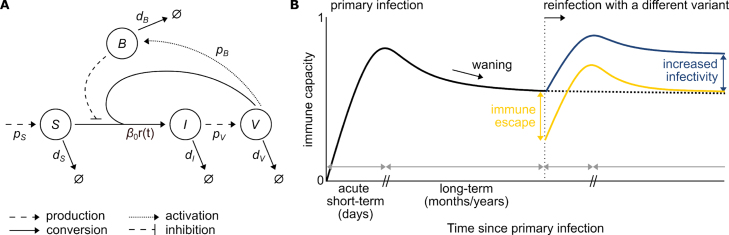
**Schematic presentation of the within-host SARS-CoV-2 infection model and diagrammatic representation of the immune capacity during primary and re-infections.** (A) Susceptible cells (S) are produced at rate $p_{S}$ and become infected at rate $\beta_{0}r\left(t\right)$, where $\beta_{0}$ is the infectivity rate, r(t)=1−B(t) is the immune capacity effect function and B(t) is the immune capacity. Infected cells (I) in turn produce free virus (V) at rate $p_{V}$. Susceptible and infected cells die at rates $d_{S}$ and $d_{I}$, respectively, and virus is cleared at rate $d_{V}$. Immune capacity B depends on the viral load V, is activated with rate $p_{B}$, and decreases with rate $d_{B}$. (B) Upon a primary SARS-CoV-2 infection, the acute short-term immune capacity detects, contains, and eliminates the virus. After viral clearance, immune protection against SARS-CoV-2 typically weakens over time. In the case of reinfection with a different variant, the immune capacity is re-activated. For a different variant with immune escape (yellow), the initial immune capacity against the different variant suffers a drop due to the variant’s escape properties. For a different variant featuring higher infectivity (blue), the immune capacity rises to higher levels in order to successfully control the infection (blue). The dotted line represents the immunecapacity without reinfection.

Our model comprises four quantities corresponding to the populations of susceptible epithelial cells (S) and infected epithelial cells (I), free virus particles (V), and the immune capacity (B, normalized to a range between 0 and 1) (Fig. 1A).

The full system of non-linear ordinary differential equations (ODEs) for the susceptible (S) and infected (I) cells, free virus (V), and the immune capacity (B) is given by the following equations:

(1) $$\frac{\partial S\left(t\right)}{\partial t}=p_{S}-d_{S}S\left(t\right)-\beta_{0}r\left(t\right)S\left(t\right)V\left(t\right)$$

$$\frac{\partial I\left(t\right)}{\partial t}=\beta_{0}r\left(t\right)S\left(t\right)V\left(t\right)-d_{I}I\left(t\right)$$

$$\frac{\partial V\left(t\right)}{\partial t}=p_{V}I\left(t\right)-d_{V}V\left(t\right)-\beta_{0}r\left(t\right)S\left(t\right)V\left(t\right)$$

$$\frac{\partial B\left(t\right)}{\partial t}=p_{B}\left(1-B\left(t\right)\right)V\left(t\right)-d_{B}\left(B\left(t\right)-B_{\text{thres}}\right)B\left(t\right),$$

where r(t)=1−B(t) is the immune capacity effect function, and $S\left(0\right)=S_{0}$, $I\left(0\right)=I_{0}$, $V\left(0\right)=V_{0}$, and B(0)=0 are the given initial values. In a disease-free system, susceptible cells are in homeostasis, where cells are constantly produced at rate $p_{S}$ and die at a natural death rate $d_{S}$, where $p_{S}=S_{0}\times d_{S}$. Once a free virus is introduced, the system is perturbed as follows: When in contact with free virus, susceptible cells get infected according to an overall infectivity rate $\beta_{0}r\left(t\right)$, which is the product of the infectivity rate constant $\beta_{0}$ and immune capacity effect function r(t). The successfully infected cells enter the infected cell population and are eliminated from that population with virus-induced death rate $d_{I}$. Due to the non-lytic property of SARS-CoV-2, infected cells constantly produce and release free virus at rate $p_{V}$, which is cleared at rate $d_{V}$ [13]. Free virus leading to successfully infected cells are lost from this population according to the overall infectivity rate $\beta_{0}r\left(t\right)$. A SARS-CoV-2 infection activates the immune response (Fig. 1B). First, the innate immune response detects the viral infection and limits its spreading within the host. The innate immune response also triggers the adaptive immune mechanisms, which ultimately clear the virus and form a lasting, intermediate SARS-CoV-2-specific immune memory [17], [18], [19]. This ensures an enhanced response to future infections of the same virus. Detectable immune response levels against SARS-CoV-2 are reported to persist for 6 to 12 months post infection [20], [21], [22]. Thereafter immune response levels drop to undetectable levels [20]. The term immune capacity B in our model represents the combined effects of innate immunity, adaptive immunity, and immune memory on the ability to control the virus. For a primary infection, the individual was assumed to not have been exposed to SARS-CoV-2 before, and hence, the immune capacity is initially described by a naive pre-infection state with B(0)=0. Upon infection, B increases proportional to the viral load at rate $p_{B}$ and is limited by the upper bound of 1, such that B(t)∈[0,1]. The activation of the immune capacity is assumed to lead to a reduced overall infectivity and hence, to fewer successful infections of susceptible cells [6], [9]. To account for the intermediate period of persisting immune capacity levels, we assumed that the immune capacity decreases to a low but detectable level of immunity after acute infection. To counteract a repeated spreading of the same virus within an individual, we defined a minimal immune threshold $B_{\text{thres}}$ against which the immune capacity B converges to in the long-term with rate $d_{B}$, while assuring a permanent clearance of the infection. The immune threshold $B_{\text{thres}}$ is given by:

(2) $$B_{\text{thres}}=1-\frac{d_{I}d_{V}}{\beta_{0}S_{0}\left(p_{V}-d_{I}\right)}.$$

How $B_{\text{thres}}$ is determined is described in detail in Section 4 Methods. We assumed that $p_{V}>d_{I}$ to assure positive overall infectivity rates. To keep the immune capacity inactive during the initial virus-free state and allow for its activation upon contact with SARS-CoV-2 only, we multiplied $d_{B}\left(B\left(t\right)-B_{\text{thres}}\right)$ by B(t) in the last equation of (1). In summary, the overall infectivity rate is

$$\beta_{0}r\left(t\right)=\left\{\begin{array}{cc} \beta_{0},\; & \;\text{if there is no immune capacity}\\ 0,\; & \;\text{if the immune capacity reaches its maximal value}\\ \beta_{0}\left(1-B_{\text{thres}}\right),\; & \;\text{if long-term immune capacity is attained}\\ \beta_{0}r\left(t\right),\; & \;\text{otherwise.} \end{array}\right)$$

After a successful infection and clearance of the virus, the susceptible cells return to the initial pre-infection level. Steady state and stability analyses demonstrate that this is the only post-disease steady state (see 4 Methods). Overall, the model is described by four quantities, S, I, V, and B, and eight parameters, $p_{S}$, $d_{S}$, $\beta_{0}$, $d_{I}$, $p_{V}$, $d_{V}$, $p_{B}$, and $d_{B}$, where all parameters are restricted to positive values for biological interpretability.

### 2.2 Infection dynamics

**Fig. 2:**
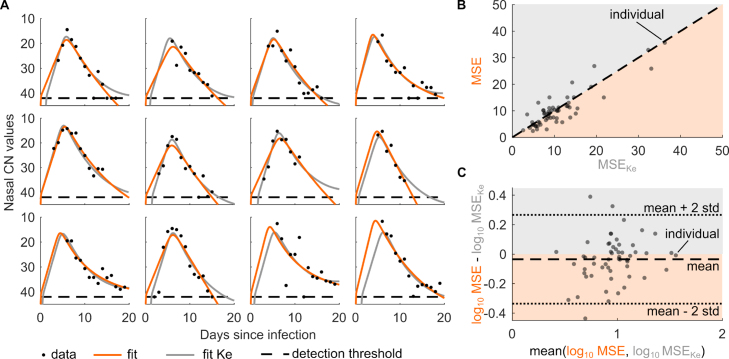
**Model captures the individual-specific viral load dynamics of a primary SARS-CoV-2 infection.** (A) Model fits of our model (orange line) and Ke model (gray line, [8]) to nasal CN values (black dots, measured by Ke et al. [8]) of 12 randomly selected individuals. The dashed line represents the detection threshold of the RT-qPCR method to determine viral presence in the nasal swab sample and is set to CN = 42. Dots on the dashed line denote measurements below the detection threshold. (B) Comparison scatter plot of the mean squared errors (MSEs) of our model fits to fits from Ke et al. (MSE${}_{\text{Ke}}$) for all 56 individuals. The dashed line indicates the diagonal representing equal MSEs between model fits. All points above the diagonal represent individuals for which the Ke model fits lead to smaller MSEs than our model (gray shaded area), while all points below the diagonal represent individuals, for which our model fits lead to smaller MSEs than the Ke model (orange shaded area). (C) Comparison of ${\text{log}}_{\text{10}}$ MSEs of model fits to all 56 individuals according to the Bland–Altman method. The mean of the ${\text{log}}_{\text{10}}$ MSE differences between our model fits and those from Ke et al. is −0.035 (black dashed line) and the mean of the ${\text{log}}_{\text{10}}$ MSE differences ±2 × standard deviations (stds) are −0.34 and 0.27, respectively (black dotted lines). All points above zero represent individuals for which the Ke model fits lead to smaller ${\text{log}}_{\text{10}}$ MSEs than our model (gray shaded area), while all points below zero represent individuals, for which our model fits lead to smaller ${\text{log}}_{\text{10}}$ MSEs than the Ke model (orange shaded area).

To quantitatively describe the dynamics of a SARS-CoV-2 infection in a human, we fitted the model to 56 individual nasal viral load samples from Ke et al. [8]. Ke et al. used reverse transcriptase polymerase chain reaction (RT-PCR) to detect SARS-CoV-2 by amplifying viral RNA in a series of repeated cycles. The number of reaction cycles needed to reach a certain cycle threshold value determines the positivity of a test and is defined as the cycle number (CN). The lower the CN value the greater the amount of viral RNA present in the original sample. To describe the transformed CN values using the model, we formally redefined population V as the virus in the nasal swab sample and, similarly, $p_{V}$ as the viral production rate times the proportion of sampled virus [6]. Accordingly, we also redefined $d_{V}$, $p_{B}$, and $\beta_{0}$ times a factor reflecting the sampling process. To account for the heterogeneity between individuals, we estimated three of the eight model rate constants, namely the viral production rate constant $p_{V}$, the activation rate constant of the immune capacity $p_{B}$, and the waning rate constant $d_{B}$, at an individual-specific level (Supplementary material). All other rate constants were assumed to be fixed and the same across individuals (Table 2). We observed a good fit of our model to the individual-specific CN values with similar dynamics as predicted by the model of Ke et al. [8] (Fig. 2A and Supplementary material). We would like to point out that Ke et al. took a non-linear mixed-effect modeling approach fitting all individuals simultaneously and estimating a total of six model parameters [8]. The main differences between model fits are in the predicted initial infection dynamics prior to peak viral loads.

From the estimated individual-specific viral production rate constants $p_{V}$ and the fixed death rate constant of an infected cell $d_{I}$, we calculated an estimated median number of 75 viral particles (interquartile range [66, 98]) being produced during the life span of an infected cell. The average produced number of viral particles per infected cell and individual is given by $\frac{p_{V}}{d_{I}}$. Furthermore, we qualitatively compared the fits of our model to those of Ke et al. [8] for all 56 individuals, using the mean squared errors (MSEs) of the respective model fits (Fig. 2B). The MSEs are closely scattered around the diagonal line, suggesting a good general agreement between the two models across individuals. For 33 of the 56 individuals, our model fits led to lower MSEs than those of Ke et al. (dots in orange area of Fig. 2B). Furthermore, we considered the Bland–Altman method for statistically assessing the agreement between the model fits [23]. Considering the log_10_ MSEs, we found a negligible bias (log_10_ MSE - log_10_ MSE${}_{\text{Ke}}$) and identified that in 95% of the individuals the MSEs of Ke et al. are between 0.46 and 1.85 times the MSEs of our model (Fig. 2C). Overall, the two models concur well in capturing the SARS-CoV-2 infection dynamics of nasal viral load samples in humans.

### 2.3 Numerical results

**Fig. 3:**
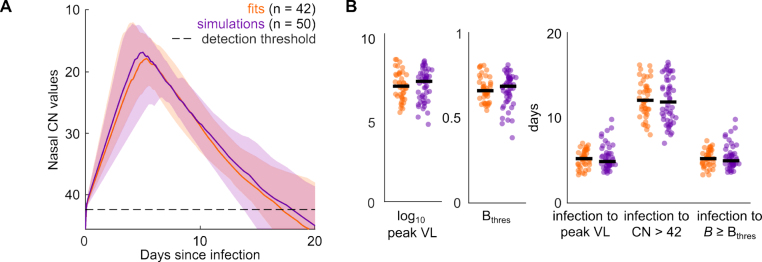
**Model reproduces key features of SARS-CoV-2 infection dynamics.** (A) Median, 10th to 90th percentiles of 42 fits of our model to nasal CN values (orange) and 50 simulated infection dynamics (purple) using our within-host model. The dashed line represents the detection threshold of the RT-qPCR method to determine viral presence in the nasal swab sample and is set to CN = 42. (B) Distributions of the five key features describing the underlying infection dynamics: (i) log_10_ peak viral loads (VL), (ii) $B_{\text{thres}}$-values and durations from (iii) infection to peak VL, (iv) peak VL to undetectable VL, defined as a CN value > 42, and (v) infection to $B>B_{\text{thres}}$ across the fits (orange) and simulations (purple). The distributions of key features across fitted and simulated infection dynamics are statistically comparable with *p*-values 0.37, 0.51, 0.42, 0.52, and 0.45, respectively (two-sample Kolmogorov–Smirnov test correcting for five-fold testing according to the Bonferroni correction). The scatter is consistent between fits and simulations across sub-panels. The median values per feature are highlighted by the black bars.

To test the generalizability of the model, we performed a simulation study. Similar to the model fitting in the previous Section 2.2 Infection dynamics, we assumed the rate constants $p_{V}$, $p_{B}$, and $d_{B}$ to be individual-specific and to capture the heterogeneity underlying the SARS-CoV-2 infection dynamics. All other rate constants were assumed to be fixed and to be the same across individuals. We drew 50 rate constant triplets out of the multivariate distribution we received from the estimated individual-specific rate constants in the previous Section 2.2 Infection dynamics. Using these sets of rate constants and our within-host model, we simulated 50 SARS-CoV-2 infection dynamics (Fig. 3A).

From all 42 fits and 50 simulations, we computed five key features describing the underlying infection dynamics, namely (i) the log_10_ peak viral load (VL), (ii) $B_{\text{thres}}$, (iii) the number of days from infection to peak VL, (iv) the number of days from peak VL to undetectable VL (defined as a CN value > 42), and (v) the number of days from infection to $B\geq B_{\text{thres}}$. We found the distributions of each of these five key features to be statistically comparable, that is we cannot reject the null hypothesis that the medians of both groups are equal, between the fitted and simulated infection dynamics with *p*-values 0.37, 0.51, 0.42, 0.52, and 0.45, respectively (Fig. 3B). The median values for each of the key features for our fitted and simulated infection dynamics are shown in Table 1 alongside those reported by earlier studies.

Our model and simulation output agree well with those reported by others. Together, the model reliably generates simulated individual-specific SARS-CoV-2 infection dynamics.

**Table 1:** Median values of key features of infection dynamics: output from our model (fitted and simulated) alongside corresponding values reported by earlier studies.

	Key features of infection dynamics	Median value			Ref.
		Fits	Simulations	Other studies	
(i)	log_10_ peak VL	7.2	7.5	4–9	[1], [4], [24]
(ii)	$B_{\text{thres}}$	0.66	0.68	–	–
(iii)	Days from infection to peak VL	5.2	4.9	4–6	[24]
(iv)	Days from peak to undetectable VL	12	12	8–11	[1], [24]
(v)	Days from infection to $B\geq B_{\text{thres}}$	5.2	5.0	–	–

### 2.4 Post-acute infection and reinfection dynamics

**Fig. 4:**
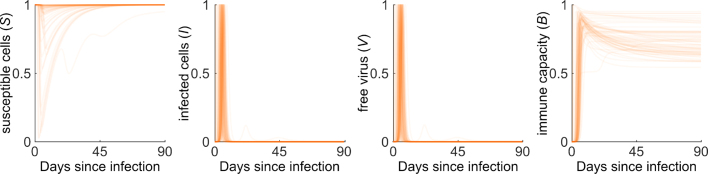
**SARS-CoV-2 infection dynamics following acute infection.** SARS-CoV-2 infection dynamics for the populations of susceptible and infected cells S and I, respectively, free virus V, and the immune capacity B for all 56 fitted individuals following acute infection. The susceptible cells are normalized per individual by the initial number of susceptible cells $S_{0}$, while the number of infected cells and free virus particles are normalized by their maximal values during infection.

To verify the model performance for biological realism of its output, we further considered our model fits of the nasal viral load data (Ke et al. [8]) for a period of 90 days post infection (Fig. 4). For all but one individual, the susceptible cells S re-establish their initial pre-infection levels after infection, which is denoted by S = 1 at 90 days since infection (Fig. 4, left panel). Moreover, the infection is permanently and fully cleared within all individuals, given by I = 0 and V = 0 after 90 days since infection (Fig. 4, middle panels). Finally, the model fits of all individuals account well for immune waning and the establishment of long-term post-acute infection immune capacity at $B_{\text{thres}}$ after infection (Fig. 4, right panel, individual-specific $B_{\text{thres}}$-values not shown). Through its intrinsic properties, our within-host SARS-CoV-2 model captures effectively the expected post-acute infection dynamics with respect to the different model compartments. As earlier models provide anomalous predicted infection dynamics when extended to a long-term post-acute infection period, e.g., a reduced number of susceptible cells post infection, a residual number of infected cells and virus leading to chronic infection, and an immune capacity which either remains at maximal levels or decreases to naive pre-infection levels, our model provides an improved reflection of the long-term post-acute infection dynamics in all four model populations.

**Fig. 5:**
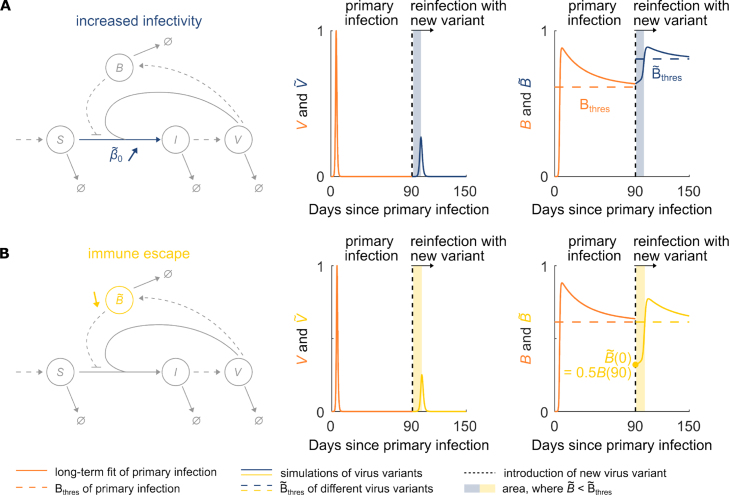
**Model fits and simulation of reinfection with virus variants with two different changes in properties: increased infectivity and immune escape.** (A) Schematic presentation of the adapted model parameterization accounting for a different virus variant with increased infectivity rate constant ${\overset{\sim }{\beta }}_{0}$ (here, ${\overset{\sim }{\beta }}_{0}=2\beta_{0}$). Model fit of the viral load V and immune capacity B during and post-acute infection of a representative individual is shown in orange. The vertical dotted black line indicates the day of contact with the respective virus variant. The continued simulation demonstrates an increase in the viral load of the variant $\overset{\sim }{V}$, and hence, reinfection (blue line). The variant-specific immune capacity dynamics of $\overset{\sim }{B}$ and corresponding variant-specific immune threshold ${\overset{\sim }{B}}_{\text{thres}}$ are shown in the right panel (blue line and blue dashed line, respectively). The blue shaded area corresponds to the duration for which $\overset{\sim }{B}<{\overset{\sim }{B}}_{\text{thres}}$ and indicates the period at which the new virus variant with increased infectivity can spread within the host. (B) Schematic presentation of the adapted model parameterization accounting for a different virus variant with immune escape reflected by a decreased initial variant-specific immune capacity $\overset{\sim }{B}$ (here, $\overset{\sim }{B}\left(0\right)=0.5B\left(90\right)$). Like in (A), the model fit of viral load V and immune capacity B during and post-acute infection of a representative individual is shown in orange. The vertical dotted black line indicates the day of contact with the respective virus variant. The continued simulation demonstrates reinfection (yellow line). The variant-specific immune capacity dynamics of $\overset{\sim }{B}$ and corresponding variant-specific immune threshold ${\overset{\sim }{B}}_{\text{thres}}$ are shown in the right panel (yellow line and dashed line, respectively). The yellow shaded area corresponds to the duration for which $\overset{\sim }{B}<{\overset{\sim }{B}}_{\text{thres}}$ and indicates the period at which the new virus variant with immune escape can spread within the host.

According to the USA Centers of Disease Control and Prevention, a reinfection is a positive detection of SARS-CoV-2 at least 90 days after a previous infection [25]. At present, reinfections typically involve a different SARS-CoV-2 variant and hence, potentially different infection dynamics due to differences in both the reinfecting variant or the variant-specific immune capacity [26]. In our model parameterization, we accounted for both of these factors separately. We explored a scenario, where the new variant features an increased infectivity, which is reflected by an increased infectivity rate constant $\beta_{0}$ in our model (Fig. 5A, left panel). Furthermore, we explored another scenario, where the new variant features immune escape. In our model, immune escape is reflected by an initial drop in the variant specific immune capacity B (Fig. 5B, left panel). We used the previously simulated post-acute primary infection dynamics of a randomly selected representative individual from the Ke et al. data set [8]. Furthermore, we assumed the individual to have been in contact with a different variant with either increased infectivity or immune escape, leading in each of these scenarios to a single successfully infected cell at 90 days since primary infection. Using the adapted variant-specific model parameterizations, that is an increased infectivity rate constant of ${\overset{\sim }{\beta }}_{0}=2\beta_{0}$ or a decreased initial immune capacity of $\overset{\sim }{B}\left(0\right)=0.5B\left(90\right)$, where ${\overset{\sim }{\beta }}_{0}$ the variant-specific infectivity rate constant and $\overset{\sim }{B}\left(t\right)$ the variant-specific immune capacity, we simulated the viral loads and immune capacities for both variants for another 60 days after reinfection (Fig. 5A and B, middle and left panels). Our corresponding simulation results show a detectable increase in the viral loads due to reinfection for both variants. As a result of pre-existing immunity from the primary infection, the immune capacity is elevated at reinfection. The full immune capacity is reached only a few days later, when the immune response is fully mounted. Given the parameterizations of ${\overset{\sim }{\beta }}_{0}$ and $\overset{\sim }{B}\left(0\right)$, the peak viral loads remain lower during reinfection than observed during primary infection for both variants. Overall, the model accommodates well for reinfection events and differentiates between reinfection modes involving distinct variant properties.

## 3 Discussion

We developed a mathematical model that describes the within-host SARS-CoV-2 infection and reinfection dynamics. Our model efficiently captures the acute short-term viral infection dynamics of the virus, and importantly, it accommodates biological phenomena surrounding the period following acute infection. By including viral loads and immune capacity dynamics during reinfection events by SARS-CoV-2 variants with increased infectivity or immune escape properties, our model accounts for aspects of the within-host infection process that go beyond current approaches.

We fitted the model to data from a previous clinical study that measured viral loads of infected individuals and found the model to describe the acute short-term infection dynamics equally well as the model fits of Ke et al. [8] (Fig. 2). We fixed all model parameters for which there are reliable value estimates available from the literature and only estimated the three rate constants for which literature yields a very wide range of possible values or no values at all. These three rate constants are the viral production rate constant and the two rates determining the dynamics of the immune capacity: the activation and the waning rate constants. A sensitivity analysis identified the performance of our model fits as highly variable to changes in the infectivity, the viral production, and the viral clearance rate constants (Supplementary material). However, our model fits performed equally well as the fits of Ke et al. [8] upon estimating the viral production rate constant only. To reduce model complexity, we hence decided against estimating the infectivity and viral clearance rate constants at an individual-specific level and fixed these rate constants to values from literature (Table 2). Small differences in the initial infection dynamics prior to peak viral loads may be the result of our model assuming that peak viral load occurs at 6 days post infection, while Ke et al. estimated the duration from infection to peak viral load [8]. As there is only little data collected before the peak viral load, it is difficult to comment on these discrepancies between models. Using our model fits, we estimated that a single infected cell produces on average 75 viral particles in total (interquartile range [66, 98]) during its life span. The number of infectious viral particles cells produce is an important property in studying intra-host viral dynamics. Our value is in the range of those reported by earlier studies: from 10 to 100 infectious particles produced by a single infected cell [27], [28].

**Fig. 6:**
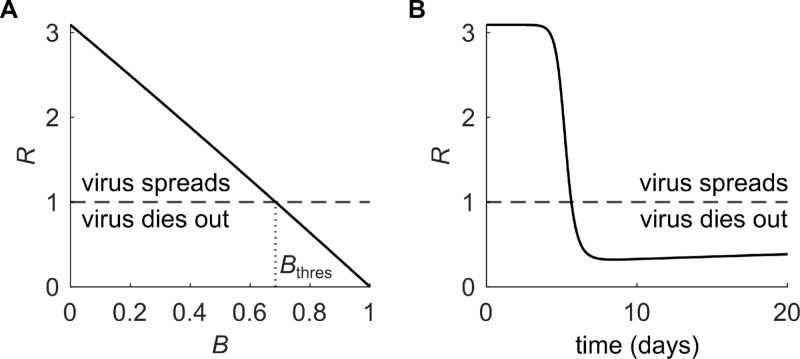
**Reproduction number R**. (A) Changes of the reproduction number R over the immune capacity B. The horizontal dashed line denotes R=1 and the vertical dotted line denotes the value of B for which R=1, defined as $B_{\text{thres}}$. For R>1, the virus spreads and for R<1 the virus dies out. (B) Changes of the reproduction number R as a function of the time-dependent immune capacity B(t) over the course of a simulated infection with parameter values from Table 2 and $p_{V}=200$, $p_{B}=10^{-8}$, and $d_{B}=10^{-2}$. The horizontal dashed line denotes R=1. At the beginning of the infection, R>1 leads to an increase in viral load. Because of the activation of the immune capacity during infection, R decreases below 1 leading to a clearance of the infection.

We embedded the overall effectiveness of the activity of the immune capacity against SARS-CoV-2 within the quantity B, representing the combined effects of innate immunity, adaptive immunity, and immune memory on the ability to control the virus. This type of approach allows for a global description of the immune response–virus interaction in tractable mathematical terms by maintaining a biological interpretation [29], [30]. According to how the immune capacity B impacts the overall infectivity rate $\beta_{0}\left(1-B\right)$ in our model, an upper bound of B is necessary to avoid negative overall infectivity rates and, hence, to ensure biological meaningfulness. Due to the time-dependent description of the immune capacity and the introduction of an immune threshold, $B_{\text{thres}}$, which is the minimal immune capacity required to counter a repeated spreading of the same virus variant within the individual, our approach provides insights into additional features regarding the course of infection within an individual. In the post-acute infection phase, the model recapitulates the restoration of the susceptible cell numbers to pre-infection homeostatic levels and accounts for the complete clearance of the infection, for which the immune threshold is critical (Fig. 4). After an acute infection, the immune capacity B converges to the immune threshold and, hence, maintains at long-term post-acute infection levels. The immune threshold in our model may reflect the persistence of the underlying immune response across sequential SARS-CoV-2 infections as observed by Kissler et al. [31]. For the definition of $B_{\text{thres}}$, we determined the basic reproduction number $R_{0}$, when no immune capacity is present, and an effective reproduction number R that is immune capacity-dependent and changes over time (Fig. 6). Stability analysis of the model allows for a lucid description of the dynamic behavior of the populations involved in the infection process and their steady states. Together, through its intrinsic properties, our within-host SARS-CoV-2 model captures effectively the expected long-term post-acute infection dynamics with respect to the different model populations. This is an improvement on earlier models, which when extended to the post-acute infection period provide anomalous predicted infection dynamics.

Our model further incorporates reinfection events by SARS-CoV-2 variants with different properties affecting within-host viral dynamics (Fig. 5). Introducing a new virus variant with increased infectivity or immune escape perturbs the convergence of B to the immune threshold by raising the threshold or by lowering the effective immunity, respectively. As a result, the immune capacity against the new virus variant at the time of secondary infection is lower than the new immune threshold (Fig. 4). According to the formal mathematical definition of the immune threshold of the virus variant ${\overset{\sim }{B}}_{\text{thres}}$ in our model, the virus variant spreads if $\overset{\sim }{B}<{\overset{\sim }{B}}_{\text{thres}}$ (Fig. 6). Only when $\overset{\sim }{B}$ reaches a value greater than ${\overset{\sim }{B}}_{\text{thres}}$ is the spread of the new virus variant contained by the new updated immune capacity $\overset{\sim }{B}$. For both kinds of new virus variants, this feature allows the model to account for the initiation of the reinfection. That ability of our model to accommodate reinfection events and differentiate between reinfection modes involving distinct variant properties allows for its applications in clinical and public health scenarios, providing a more realistic description of key outcomes.

This study has several limitations. In contrast to other existing models, our within-host SARS-CoV-2 model does not account for the time delay between a cell being infected and it becoming infectious, i.e., actively producing virus, termed eclipse phase (Fig. 1A). Considering that the acute infection is on average 20 days [32], an eclipse period of about 6 h [6] is short in comparison. Hence, we do not expect the omission of the eclipse phase to change the results qualitatively. Moreover, the immune capacity in the model is summarized by the dynamic variable B, which regulates the overall infectivity. There is a large variation on how modeling approaches incorporate the immune response and its effects on the viral dynamics of SARS-CoV-2 throughout literature [4], [6], [8], [9]. Our model uses an approach, which properly captures more of the dynamic features of the immune capacity, such as waning immunity, while at the same time describing the short-term viral load dynamics as clinically observed (Fig. 2A). Therefore, we believe our model assumptions to be justified. Furthermore, our aim was to develop a within-host SARS-CoV-2 model, which not only describes the acute infection phase, but also the post-acute infection dynamics and reinfection. We therefore assumed a simple optimization approach for describing the individual viral load dynamics and performed a qualitative comparison between our model fits and the original fits of Ke et al. [8] (Fig. 2). Further, it must be noted that the possibility of reinfection with the same virus variant is not accounted for in the model. While, in theory this is possible as a result of waning immunity, in reality the slow nature of waning combined with the rapid evolution of SARS-CoV-2 makes is unlikely for the variant causing the primary infection to be around by the time immunity is low enough to allow reinfection with it. Finally, we would like to highlight that we did not validate the model simulations for the long-term post-acute infection and reinfection dynamics due to the lack of data.

Although developed for the infection of cells in the upper respiratory tract the model can be easily adapted to account for the lower respiratory tract and extended to assess the effects of treatment options and drug resistance development.

Overall, the model presents an advance in the description of the SARS-CoV-2 infection dynamics within humans capturing the full dynamic infection process from initial infection to the clearance of the virus to reinfection and the corresponding short-term and persisting immune capacity across multiple infections. This modeling approach should be of interest for clinical use when quantitatively describing the within-host SARS-CoV-2 infection and developing treatment options seeking optimal treatment design.

## 4 Methods

### 4.1 Determining immune threshold $B_{\text{thres}}$

We defined immune threshold $B_{\text{thres}}$ as the minimal immune capacity required to counter a repeated spreading of the same virus variant within an individual. To determine $B_{\text{thres}}$, we first derived the basic reproduction number $R_{0}$, which signifies the number of secondary virus particles generated by the infection of a single virus when introduced into a fully susceptible cell population, hence, $S=S_{0}$. If $R_{0}>1$, more virus particles are generated each generation leading to an increase of the viral population. If $R_{0}<1$, less virus particles are generated each generation letting the infection in the individual run out over time. To calculate $R_{0}$, we first determined the Jacobian of the infected subsystem including compartments I and V:

$$J_{\text{sub}}=\left(\begin{array}{cc} -d_{I} & \beta_{0}rS_{0}\\ p_{V} & -d_{V}-\beta_{0}rS_{0} \end{array}\right)=\underset{≔T}{\underset{︸}{\left(\begin{array}{cc} 0 & \beta_{0}rS_{0}\\ 0 & 0 \end{array}\right)}}+\underset{≔\Sigma }{\underset{︸}{\left(\begin{array}{cc} -d_{I} & 0\\ p_{V} & -d_{V}-\beta_{0}rS_{0} \end{array}\right)}},$$

where T corresponds to the transmission and Σ to the transition matrix [33]. The next-generation matrix NGM is then given by

$$\text{NGM}=-T\Sigma^{-1}=\frac{1}{d_{I}\left(d_{V}+\beta_{0}rS_{0}\right)}\left(\begin{array}{cc} p_{V}\beta_{0}rS_{0} & d_{I}\beta_{0}rS_{0}\\ 0 & 0 \end{array}\right),$$

whose dominant eigenvalue is the reproduction number R, where R is dependent on B and hence time t (Fig. 6):

(3) $$R=\frac{p_{V}\beta_{0}rS_{0}}{d_{I}\left(d_{V}+\beta_{0}rS_{0}\right)}=\frac{p_{V}\beta_{0}\left(1-B\right)S_{0}}{d_{I}\left(d_{V}+\beta_{0}\left(1-B\right)S_{0}\right)}.$$

The reproduction number R can be split into

$$R=\frac{p_{V}\beta_{0}rS_{0}}{d_{I}\left(d_{V}+\beta_{0}rS_{0}\right)}=\underset{\text{(i)}}{\underset{︸}{\frac{p_{V}}{d_{I}}}}\underset{\text{(ii)}}{\underset{︸}{\frac{\beta_{0}rS_{0}}{\left(d_{V}+\beta_{0}rS_{0}\right)}}},$$

where (i) represents the number of viral particles produced during the life span of an infected cell and (ii) denotes the number of susceptible cells infected during the life span of a free viral particle. At the time of infection, the immune capacity is naive, such that the initial reproduction number $R_{0}$ without immune capacity (B=0) is given by:

$$R_{0}=\frac{p_{V}\beta_{0}S_{0}}{d_{I}\left(d_{V}+\beta_{0}S_{0}\right)}.$$

The value $B_{\text{thres}}$ is then determined so that R=1 for $B=B_{\text{thres}}$. In the case $p_{V}\leq d_{I}$, it is easy to show that R<1 for any B∈[0,1]. This means that infection is not possible in this case, therefore we further assume that $p_{V}>d_{I}$. Assuming the susceptible cells S reach their homeostatic level $S_{0}$ at the end of an infection, $B_{\text{thres}}$ in ODE system (1) is given by

(4) $$B_{\text{thres}}=1-\frac{d_{I}d_{V}}{\beta_{0}S_{0}\left(p_{V}-d_{I}\right)}.$$

We assume $B_{\text{thres}}>0$, to ensure the possibility of infection within the individual.

### 4.2 Steady states

The steady states are defined as the states for which the right-hand sides of the ODE system (1) are all equal to zero. We directly see that the pre-infection steady state is given by $\left(S^{*},I^{*},V^{*},B^{*}\right)=\left(S_{0},0,0,0\right)$. To identify the stability of the disease-free steady state, we first considered the Jacobian of the full ODE system described by (1),

(5) $$J=\left(\begin{array}{cccc} -d_{S}-\beta_{0}\left(1-B\right)V & 0 & -\beta_{0}\left(1-B\right)S & \beta_{0}SV\\ \beta_{0}\left(1-B\right)V & -d_{I} & \beta_{0}\left(1-B\right)S & -\beta_{0}SV\\ -\beta_{0}\left(1-B\right)V & p_{V} & -d_{V}-\beta_{0}\left(1-B\right)S & \beta_{0}SV\\ 0 & 0 & p_{B}\left(1-B\right) & -p_{B}V-2d_{B}B+d_{B}B_{\text{thres}}, \end{array}\right)$$

and determined the Jacobian at the disease-free steady state:

$$J{|}_{\left(S^{*},I^{*},V^{*},B^{*}\right)}=\left(\begin{array}{cccc} -d_{S} & 0 & -\beta_{0}S_{0} & 0\\ 0 & -d_{I} & \beta_{0}S_{0} & 0\\ 0 & p_{V} & -d_{V}-\beta_{0}S_{0} & 0\\ 0 & 0 & p_{B} & d_{B}B_{\text{thres}} \end{array}\right).$$

We then identified the eigenvalues from

$$\text{det}\left(J{|}_{\left(S^{*},I^{*},V^{*},B^{*}\right)}-\lambda I\right)=\left(-d_{S}-\lambda \right)\left(-d_{I}-\lambda \right)\left(-d_{V}-\beta_{0}S_{0}-\lambda \right)\left(d_{B}B_{\text{thres}}-\lambda \right)-\;\left(-d_{S}-\lambda \right)p_{V}\beta_{0}S_{0}\left(d_{B}B_{\text{thres}}-\lambda \right)=0,$$

with $\lambda_{1}=-d_{S}<0$ and $\lambda_{2}=d_{B}B_{\text{thres}}>0$. From the remaining quadratic equation

$$\lambda^{2}+\underset{≔b^{*}}{\underset{︸}{\left(d_{I}+d_{V}+\beta_{0}S_{0}\right)}}\lambda +\underset{≔c^{*}}{\underset{︸}{d_{I}d_{V}+\left(d_{I}-p_{V}\right)\beta_{0}S_{0}}},$$

where $c^{*}<0$, as $\frac{d_{I}d_{V}}{\beta_{0}S_{0}\left(p_{V}-d_{I}\right)}<1$ according to the definition of $B_{\text{thres}}$. Then the eigenvalues are $\lambda_{3,4}=\frac{-b^{*}\pm \sqrt{b^{*2}-4a^{*}c^{*}}}{2a^{*}}$, with $a^{*}=1$, and we get $\lambda_{3}<0$ and $\lambda_{4}>0$. Overall, this signifies that the disease-free steady state is unstable. Any initial amount of virus activates the immune response B, which then approaches $B_{\text{thres}}$. Similarly, upon successful infection and recovery the post-infection steady state is given by $\left(S^{**},I^{**},V^{**},B^{**}\right)=\left(S_{0},0,0,B_{\text{thres}}\right)$, where the immune capacity reaches memory $B_{\text{thres}}$ countering reinfection of the same virus variant. The Jacobian at $\left(S^{**},I^{**},V^{**},B^{**}\right)$ is given by

$$J{|}_{\left(S^{**},I^{**},V^{**},B^{**}\right)}=\left(\begin{array}{cccc} -d_{S} & 0 & -\beta_{0}\left(1-B_{\text{thres}}\right)S_{0} & 0\\ 0 & -d_{I} & \beta_{0}\left(1-B_{\text{thres}}\right)S_{0} & 0\\ 0 & p_{V} & -d_{V}-\beta_{0}\left(1-B_{\text{thres}}\right)S_{0} & 0\\ 0 & 0 & p_{B}\left(1-B_{\text{thres}}\right) & -d_{B}B_{\text{thres}} \end{array}\right),$$

with

$$\text{det}\left(J{|}_{\left(S^{**},I^{**},V^{**},B^{**}\right)}-\lambda I\right)=\left(-d_{S}-\lambda \right)\left(-d_{I}-\lambda \right)\left(-d_{V}-\beta_{0}\left(1-B_{\text{thres}}\right)\times \;\;S_{0}-\lambda \right)\left(-d_{B}B_{\text{thres}}-\lambda \right)-\left(-d_{S}-\lambda \right)p_{V}\beta_{0}\times \;\;\left(1-B_{\text{thres}}\right)S_{0}\left(-d_{B}B_{\text{thres}}-\lambda \right)=0.$$

From this equation we get eigenvalues $\lambda_{1}=-d_{S}<0$ and $\lambda_{2}=-d_{B}B_{\text{thres}}<0$. For eigenvalues $\lambda_{3,4}$ we need to solve the remaining quadratic equation

$$\lambda^{2}+\left(d_{V}+\beta_{0}\left(1-B_{\text{thres}}\right)S_{0}+d_{I}\right)\lambda +\underset{≔c^{**}}{\underset{︸}{d_{I}d_{V}+\left(d_{I}-p_{V}\right)\left(\beta_{0}\left(1-B_{\text{thres}}\right)S_{0}\right)}}=0.$$

When inserting $B_{\text{thres}}$ from (4), we see that $c^{**}=0$ and hence, $\lambda_{3}=-\left(d_{V}+\beta_{0}\left(1-B_{\text{thres}}\right)S_{0}+d_{I}\right)<0$ and $\lambda_{4}=0$. Thus, the investigation of stability of the steady state $X^{**}≔\left(S_{0},0,0,B_{\text{thres}}\right)$ is not determined by its linearization and requires higher order analysis. According to the center manifold theorem (see e.g. Theorem 5.1 in [34]), there is an one-dimensional invariant manifold (a curve) C passing through $X^{**}$ tangent to the eigenvector $l_{\left(\lambda_{4}=0\right)}$, corresponding to the eigenvalue $\lambda_{4}=0$. The stability of the whole system is determined by its stability on the curve C. On this curve, the system can be either be instable, semi-stable (on one part of the curve only) or stable. The eigenvector $l_{\left(\lambda_{4}=0\right)}$ is given by

$$l_{\left(\lambda_{4}=0\right)}=\left(-\frac{d_{I}d_{V}}{d_{S}},\;d_{V},\;p_{V}-d_{I},\;\frac{p_{B}\left(1-B_{\text{thres}}\right)\left(p_{V}-d_{I}\right)}{d_{B}B_{\text{thres}}}\right).$$

The analytical description of the center manifold and the investigation of its stability is non-trivial. Hence, we rely on the numerical results, which clearly show local stability of system (1). In such a case, C is a so-called “slow manifold”: the convergence to the steady state is not exponential but is at most so fast as $1/\sqrt{t}$ when time t increases to infinity. This is also consistent with the numerical results. There exists a third unique steady-state, however, not within the positive orthant, which is invariant. Since all eigenvalues are real and the trajectories of the system converge locally to the steady state $X^{**}$, the latter is globally stable in the positive orthant. Generally, this steady state can be approached asymptotically in one of two ways: either from above, when the short-term acute immune capacity is strong enough to drive $B\left(t\right)>B_{\text{thres}}$ in the initial phase of infection or from below. From the ODE system (1) we see that $\frac{\partial B}{\partial t}=0$ when

(i) $\left(\overset{¯}{V},\overset{¯}{B}\right)=\left(0,B_{\text{thres}}\right)$
(ii) $\left(\overset{¯}{\overset{¯}{V}},\overset{¯}{\overset{¯}{B}}\right)=\left(\frac{d_{B}\left(\overset{¯}{\overset{¯}{B}}-B_{\text{thres}}\right)\overset{¯}{\overset{¯}{B}}}{p_{B}\left(1-\overset{¯}{\overset{¯}{B}}\right)},\overset{¯}{\overset{¯}{B}}\right)$.

For the latter to make sense biologically $\overset{¯}{\overset{¯}{V}}\geq 0$, and hence it is required that $\overset{¯}{\overset{¯}{B}}\geq B_{\text{thres}}$. Whether the short-term acute immune capacity is strong enough to lead to $B\geq B_{\text{thres}}$ depends on the viral load V and parameter $p_{B}$. The definitions of B and $B_{\text{thres}}$ assure that the infection is fully and permanently cleared in the model, which is in line with current literature on SARS-CoV-2 for non-immunocompromised individuals. Without immune threshold at $B_{\text{thres}}$, the model would lead to a minimal but chronic infection with periodic spreading of the virus within the individual.

### 4.3 Data and pre-processing of single-individual SARS-CoV-2 infection dynamics

During fall 2020 and spring 2021, Ke et al. collected daily nasal samples for up to 14 days of all faculty, staff and students of the University of Illinois at Urbana-Champaign, who either (i) reported a positive quantitative reverse transcription polymerase chain reaction (RT-qPCR) result in the past 24 h or (ii) were within five days of exposure to someone with a confirmed positive RT-qPCR result, while having tested negative for SARS-CoV-2 in the previous seven days [8]. These criteria ensured a large-scale, high-frequency screening of early SARS-CoV-2 infection dynamics. In total, Ke et al. reported the cycle number (CN) values of nasal swab samples over time for 60 individuals. Due to very low or undetectable viral loads, we removed four out of the 60 individuals prior to the analysis, as also done by Ke et al. Time was reported relative to the day at which the maximal CN value was measured. The model is, however, initialized at infection. To compare measured and simulated CN values on the same time scale, we assumed viral load to peak six days post infection, as reported throughout literature for early SARS-CoV-2 variants [24], [35]. Hence, we shifted the relative time scale of the CN values measured by Ke et al. by six days and removed all measurements prior to the assumed day of infection. Moreover, to directly compare CN values and simulated viral loads, we made use of the CN value-to-viral load calibration determined by Ke et al. [8] and given by

(6) $$\text{CN}=-\frac{{\text{log}}_{10}V-11.35}{0.25}.$$

### 4.4 Parameterization of the within-host SARS-CoV-2 model

To describe the individual SARS-CoV-2 infection dynamics, we parameterized the model as shown in Table 2.

Initial values and the rate constants for five out of the eight parameters were taken from literature and assumed to be the same across individuals. The initial number of susceptible cells $S_{0}$ was derived by Ke et al. [8]. Otherwise, we assumed the system to be initialized by a single successfully infected cell. Assuming that the system was in equilibrium during the initial disease-free state, we set the susceptible cell production rate constant $p_{S}$ to $S_{0}\times d_{S}$, such that the in- and outflow of S is the same. The rate constant values for the infection rate $\beta_{0}$ and the death rate of an infected cell $d_{I}$ were taken from Ke et al. and are the mean rate constants of their estimated individual-level rate constants [8]. In the absence of an acute viral infection, the death rate constant of a susceptible cell $d_{S}$ is low, measured in the order of 0.02–0.03 /day [36]. However, this rate constant is estimated to increase during acute infection [12]. For simplicity, we assumed a constant death rate constant between the lower and upper estimates. The value for the viral clearance rate constant $d_{V}$ was adopted from other studies [37], [38]. To account for the heterogeneity between individuals, we estimated the other three rate constants, namely (i) the viral production rate constant $p_{V}$, (ii) activation rate constant of the immune capacity $p_{B}$, and (iii) waning rate constant of the immune capacity $d_{B}$ at an individual-specific level. The upper and lower boundaries of $p_{V}$ were determined by the range of estimated individual-specific rate constants from Ke et al. [8]. The lower and upper boundaries of $p_{B}$ and $d_{B}$ are less interpretable and, hence, were assumed to cover a broad range of values.

**Table 2:** Variables and rate constants of the model to describe the individual nasal viral load samples from Ke et al. [8]. Rate constants with ${}^{*}$ were estimated within the given upper and lower boundaries.

Variable		Initial value	Ref.
S	Number of susceptible cells	$S_{0}=8\times 10^{7}$	[8]
I	Number of infected cells	$I_{0}=1$	–
V	Number of measured virus	$V_{0}=0$	–
B	Relative immune response	$B_{0}=0$	–

Rate constant		Value	Ref.

$p_{S}$	Susceptible cell production rate constant	$S_{0}\times d_{S}$ /day	–
$d_{S}$	Death rate constant of a susceptible cell	0.091 /day	[12], [36]
$\beta_{0}$	Infectivity rate constant	$4.92\times 10^{-9}$ /day	[8]
$d_{I}$	Death rate constant of an infected cell	2.45 /day	[8]
$p_{V}^{*}$	Viral production rate constant of infected cell ×sampled virus	$\left[10^{2},10^{3}\right]$ /day	–
$d_{V}$	Viral clearance rate constant	10 /day	[37], [38]
$p_{B}^{*}$	Activation rate constant of immune capacity	$\left[10^{-10},10^{-4}\right]$ /day	–
$d_{B}^{*}$	Waning rate constant of immune capacity	$\left[10^{-4},1\right]$ /day	–

### 4.5 Parameter estimation

Experimental data such as the measurements of CN values is noise corrupt. We took this measurement noise into account in the model, by assuming an additive Gaussian measurement noise distribution. The log-likelihood for the Gaussian noise model, for individual i, time point k with measured CN value ${\overset{¯}{y}}_{i}^{k}$ is given by

$$\text{logL}\left(\theta_{i}\right)=-\frac{1}{2}\underset{k}{\sum }\text{log}\left(2\pi \sigma_{i}^{2}\right)+\frac{{\left({\overset{¯}{y}}_{i}^{k}-y\left(t_{k},\theta_{i}\right)\right)}^{2}}{\sigma_{i}^{2}}.$$

Hence, we also inferred an individual-specific noise parameter $\sigma_{i}$ per individual determining the spread of the Gaussian noise model. The lower and upper boundaries of σ were set to $\left[10^{-2},10\right]$ according to the range of CN values measured. In total we estimated four individual-specific model parameters, $p_{V}$, $p_{B}$, $d_{B}$, and σ. We performed multi-start maximum likelihood optimization of the negative log-likelihood in the log_10_ parameter space for numerical reasons [39], initiating the optimization runs from 10 different Latin-hypercube-sampled starting points, maximizing over the CN values per individual. We used the MATLAB functions *fmincon* for optimization [40] and *ode45* for solving ODEs [41]. All estimated individual-specific model parameters are summarized in the supplementary material.

### 4.6 Numerical results

The simulation study is based on the parameterization given in Table 2. Based on the single-individual model fitting, we assumed five of the eight rate constants to be the same across all individuals, namely $p_{S}$, $d_{S}$, $\beta_{0}$, $d_{I}$, and $d_{V}$, while the remaining three rate constants, $p_{V}$, $p_{B}$, and $d_{B}$, were assumed to be individual-specific. These three rate constants were previously estimated for each of the 56 individuals measured by Ke et al. [8] by using our model (Fig. 3A). For the simulation study, we removed 14 out of the 56 individuals for which the estimated standardized rate constants of $p_{V}$, $p_{B}$, and $d_{B}$ were not within ±2 × standard deviations (stds) of their respective standardized estimated distributions. For these individuals, the CN nasal swab samples have either not been collected during early infection, such that infection dynamics before peak viral load are not well captured by the model, or where the CN values did not include sufficient information to deduce the dynamics of long-term immune capacity formation. We ensured that each of the three remaining standardized distributions were standard normally distributed by performing a one-sample Kolmogorov–Smirnov test employing the *kstest* function of MATLAB [42]. The function *kstest* tests the null hypothesis that the parameter sample is drawn from a standard Gaussian distribution. As we required all three statistical tests to not reject the null hypothesis, we corrected for three-fold testing by applying the Bonferroni correction and adjusted the significance level of 0.05 to $\frac{0.05}{3}=0.0167$ [43]. The *p*-values for the standardized estimated distributions of $p_{V}$, $p_{B}$, and $d_{B}$ are 0.48, 0.99, and 0.19, respectively. By drawing from this standardized multivariate-Gaussian distribution, we maintained the correlation between the parameters as estimated previously and assured that their values were within a plausible range. In total, we drew 50 rate constant triplets out of the resulting standardized multivariate-Gaussian distribution and used these re-transformed rate constants to simulate 50 nasal viral infection dynamics for up to 20 days post infection (Fig. 3B).

### 4.7 Comparison of key features

We compared five key features of the fitted and simulated infection dynamics, namely (i) the peak viral load, (ii) $B_{\text{thres}}$, (iii) the number of days from infection to peak viral load, (iv) the number of days from peak viral load to undetectable viral load, and (v) the number of days from infection to $B\geq B_{\text{thres}}$ (Fig. 3B). For the fits and simulations which did not reach undetectable CN values or for which $B≱B_{\text{thres}}$ before 20 days post infection, we set these values to the maximum of 20 days. For each of the key features, we compared the resulting distributions from the fitted and simulated infection dynamics by performing a two-sample Kolmogorov–Smirnov test as provided by MATLAB function *kstest2*. The function *kstest2* tests for the null hypothesis that both parameter samples come from the same continuous distribution. As we tested for a single universal null hypothesis, where all five alternative null-hypotheses were required to not be rejected, we corrected for five-fold testing by applying the Bonferroni correction and adjusting the significance level of 0.05 to $\frac{0.05}{5}=0.01$ [43].

### 4.8 Reinfection

We differentiated between two different variants employing distinct modes of reinfection: (i) increased infectivity or (ii) immune escape. Increased infectivity was introduced into the model by an increased infection rate constant ${\overset{\sim }{\beta }}_{0}>\beta_{0}$ and $B_{\text{thres}}$ was modified to

(7) $${\overset{\sim }{B}}_{\text{thres}}=1-\frac{d_{I}d_{V}}{{\overset{\sim }{\beta }}_{0}S_{0}\left(p_{V}-d_{I}\right)},$$

according to its definition (Eq. (4), Fig. 5A). If ${\overset{\sim }{\beta }}_{0}>\beta_{0}$, then ${\overset{\sim }{B}}_{\text{thres}}>B_{\text{thres}}$. Similarly, immune escape was introduced into the model by an initially decreased level of the variant-specific immune capacity, $\overset{\sim }{B}\left(0\right)<B\left(\overset{\sim }{t}\right)$, where $\overset{\sim }{B}$ is the immune capacity of the new virus variant and $\overset{\sim }{t}$ is the time of reinfection (Fig. 5B). For this virus variant, the immune threshold remains the same, such that ${\overset{\sim }{B}}_{\text{thres}}=B_{\text{thres}}$. For the simulations, we assumed individual #432192 to have been exposed to one of the variants, leading to a single successfully infected cell at 90 days post primary infection. We then simulated the ODE system of (1) for the new virus variant for another 60 days. The initial values of the number of susceptible cells S and measured virus V, as well as the immune capacity B for the simulations of reinfection, were taken from the long-term fit of individual #432192 at 90 days since primary infection. We set the increased infection rate arbitrarily to ${\overset{\sim }{\beta }}_{0}=2\beta_{0}$ and immune escape to 50%, such that $\overset{\sim }{B}\left(0\right)=0.5B\left(\overset{\sim }{t}\right)$.

### 4.9 Implementation and code availability

There is no original data underlying this work. Only previously published data was used for this study [8]. The MATLAB code corresponding to this manuscript will be made available upon acceptance of the manuscript. The analysis was performed with MATLAB 2023a.

## CRediT authorship contribution statement

**Lea Schuh:** Writing – review & editing, Writing – original draft, Visualization, Validation, Software, Methodology, Investigation, Formal analysis, Data curation, Conceptualization. **Peter V. Markov:** Writing – review & editing. **Vladimir M. Veliov:** Writing – review & editing, Writing – original draft, Formal analysis. **Nikolaos I. Stilianakis:** Writing – review & editing, Writing – original draft, Supervision, Resources, Project administration, Methodology, Conceptualization.

## Declaration of competing interest

None.

## Data Availability

There is no original data underlying this work. The MATLAB code corresponding to this manuscript will be made available on GitHub upon acceptance of the manuscript.

## Appendix A Supplementary data

MMC S1 Supplementary material including the estimated rate constants, model fits, and sensitivity analysis.
