# Supplementary material for: A mathematical model for the within-host (re)infection dynamics of SARS-CoV-2
Source: Math Biosci. 2024 May;371:None. doi: 10.1016/j.mbs.2024.109178 (PMC11636724; doi:10.1016/j.mbs.2024.109178)
Supplement: MMC S1 — Supplementary material including the estimated rate constants, model fits, and sensitivity analysis. [file mmc1.pdf]

Supplementary material to "A mathematical model for the within-host (re)infection dynamics of SARS-CoV-2"

Lea Schuh, Peter V. Markov, Vladimir M. Veliov, Nikolaos I. Stilianakis

1 Estimated individual-specific rate constants

#	ID	p_V (/day)	$p_B(10^{-6}$/day)	d_B (/day)	σ
1	432192	163.67	0.2406	0.0434	3.2
2	432568	146.42	0.8943	0.0495	2.7
3	432662	178.93	0.2669	0.0645	3.0
4	432686	241.87	0.1656	0.1433	3.2
5	432864	197.69	0.0158	0.0897	2.1
6	432870	151.97	0.8226	0.0269	3.2
7	433227	151.99	0.1547	0.0378	2.9
8	433409	208.53	0.0628	0.0001	2.2
9	435729	214.57	0.1231	0.1661	2.2
10	435772	164.04	0.1034	0.0001	4.5
11	435786	237.78	0.0329	0.1788	6.0
12	435794	250.43	0.0061	0.1187	3.3
13	435805	323.89	0.0005	0.6095	2.7
14	435933	166.97	0.6042	0.0001	3.3
15	435985	190.03	0.0699	0.0755	3.9
16	437388	150.92	1.3975	0.0869	2.4
17	438577	285.62	0.0136	0.0919	2.4
18	441582	171.69	0.3965	0.0696	3.0
19	441736	192.95	0.0637	0.0714	1.5
20	441925	176.88	0.0541	0.0784	1.6
21	442978	528.77	0.0004	0.4909	2.7
22	443108	175.14	0.0280	0.0304	1.7
23	443176	153.30	2.7744	0.1153	2.3
24	444316	184.89	1.0057	0.1430	4.4
25	444332	154.01	0.0814	0.2218	5.7
26	444349	175.90	0.2507	0.0001	3.0
27	444391	178.30	0.009	0.0575	2.0
28	444446	325.59	0.0058	0.1441	3.0
29	444633	215.72	0.0753	0.0937	3.1
30	445534	999.98	0.3653	0.1574	5.1
31	445535	158.03	0.0080	0.0001	2.4
32	445602	177.54	0.3006	0.0920	3.2
33	446645	114.72	1.7813	0.0001	3.9
34	449507	138.31	1.2784	0.0001	2.8
35	449527	190.15	42.3557	0.4875	3.4
36	449614	248.00	0.9939	0.1576	3.4
37	450241	166.84	0.0070	0.0670	2.7
38	450253	192.31	0.6982	0.0501	3.9
39	451152	149.94	0.2499	0.0454	3.4
40	451231	199.43	0.1399	0.0724	3.2

#	ID	p_V (/day)	$p_B (10^{-6}$/day)	d_B (/day)	σ
41	451346	184.99	0.1617	0.1194	2.4
42	451479	312.83	0.0492	0.1156	3.2
43	451709	138.77	1.2140	0.2014	2.1
44	453058	268.18	0.0008	0.0001	1.7
45	459589	177.92	0.0977	0.0227	2.2
46	459597	197.89	0.1648	0.0049	3.6
47	461379	209.06	0.0208	0.0036	3.3
48	461764	308.60	0.0019	0.0978	2.7
49	461771	168.71	0.0499	0.0646	2.5
50	469115	151.03	0.4112	0.0574	3.0
51	470855	122.27	4.9885	0.0001	5.2
52	471467	147.32	0.6441	0.0099	3.2
53	471588	207.15	0.0218	0.1701	3.6
54	471890	485.69	0.0005	0.2597	3.4
55	473097	321.73	0.0026	0.1003	1.9
56	473107	266.48	0.0085	0.0636	3.9

Table 1: **Estimated individual-specific rate constants of viral production p_V, activation and decrease of the immune capacity p_B and d_B, respectively, and the noise parameter σ.** The ID's per individual are given according to their assignment by Ke et al., 2022.

2 Model fits of all individuals

3 Sensitivity Analysis

To identify how changes in specific parameter values affect our model fit, we performed a sensitivity analysis. For this, we considered all independent fixed (d_S, β_0, d_I, and d_V) and estimated (p_V, p_B, and d_B) rate constants of a random, representative individual. We let each rate constant vary between 0.9 and 1.1 of its original value. To receive the profile likelihoods, we re-calculated the log-likelihoods of the model to the viral load data, while altering each individual rate constant separately across 0.9 and 1.1 of its original value (Figure 2).

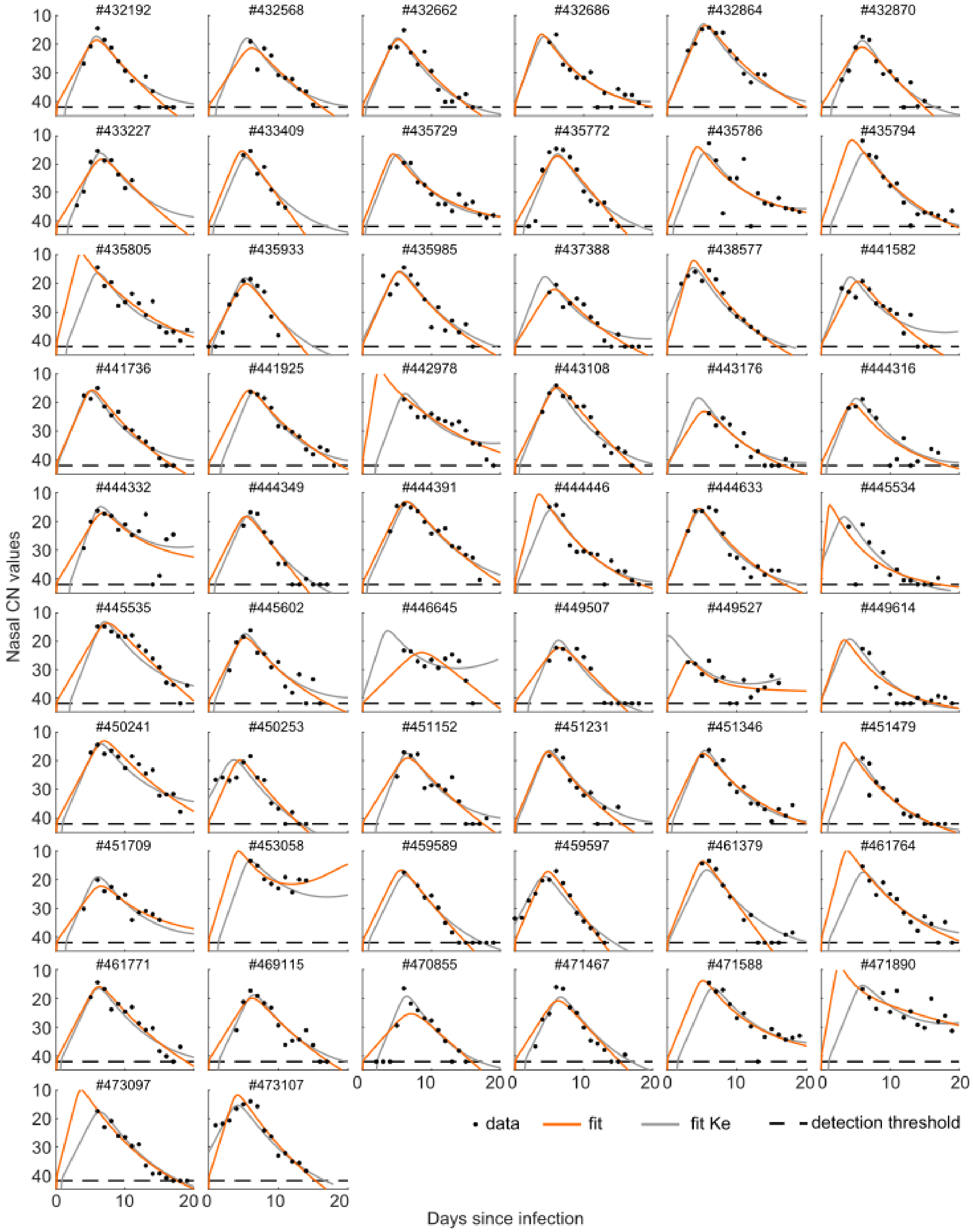

Figure 1: **Model fits of all individuals.** Model fits of our model (orange line) and Ke model (gray line, Ke et al., 2022) to nasal CN values (black dots, measured by Ke et al., 2022) of all 56 individuals. The dashed line represents the detection threshold of the RT-qPCR method to determine viral presence in the nasal swab sample and is set to CN = 42. Dots on the dashed line denote measurements below the detection threshold.

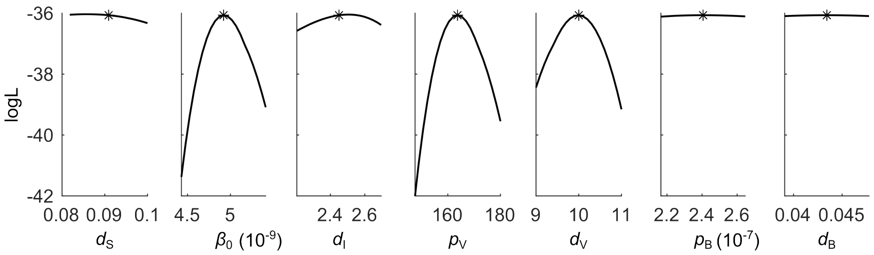

Figure 2: **Sensitivity analysis.** Profile likelihoods of the model for a random, representative individual. Asterisks represent the fixed or optimized parameters and their corresponding log-likelihood (logL) values.
